# Optimizing Flame Retardancy and Durability of Melamine-Formaldehyde/Solid-Urban-Waste Composite Panels

**DOI:** 10.3390/polym13050712

**Published:** 2021-02-26

**Authors:** Carola Esposito Corcione, Francesca Ferrari, Raffaella Striani, Laura Dubrulle, Paolo Visconti, Mauro Zammarano, Antonio Greco

**Affiliations:** 1 Department of Engineering for Innovation, University of Salento, 73100 Lecce, Italy; francesca.ferrari@unisalento.it (F.F.); raffaella.striani@unisalento.it (R.S.); paolo.visconti@unisalento.it (P.V.); antonio.greco@unisalento.it (A.G.); 2 National Institute of Standards and Technology NIST, 100 Bureau Drive, Gaithersburg, MD 20899, USA; laura.dubrulle@nist.gov (L.D.); mauro.zammarano@nist.gov (M.Z.)

**Keywords:** solid urban waste, melamine-formaldehyde, valorization process, fire retardant, durability

## Abstract

In our previous study, an innovative method for sterilization, inertization, and valorization of the organic fraction of municipal solid waste (OFMSW), to be recycled in the production of composite panels, was developed. In this follow-up work, the effects of fire retardants on fire performance, durability, and the mechanical properties of the composite panels based on OFMSW and melamine-formaldehyde resin were investigated. The performance of panels without fire retardants (control panels) was compared to panels containing either mono-ammonium phosphate (PFR) or aluminium trihydrate (ATH) at a mass fraction of 1% and 10% (modified panels). As shown by cone calorimetry, the total heat released was already low (about 31 MJ/m^2^ at 50 kW/m^2^) in the control panels, further decreased in the modified panels with the addition of fire retardants, and reached the lowest value (about 1.4 MJ/m^2^) with 10% mass fraction of PFR. Hence, the addition of fire retardants had a beneficial effect on the response to fire of the panels; however, it also reduced the mechanical properties of the panels as measured by flexural tests. The deterioration of the mechanical properties was particularly obvious in panels containing 10% mass fraction of fire retardants, and they were further degraded by artificial accelerated weathering, carried out by boiling tests. Ultimately, the panels containing PFR at a mass fraction of 1% offered the best balance of fire resistance, durability, and mechanical performance within the formulations investigated in this study.

## 1. Introduction

Recently, municipal solid waste re-utilization has become one of the main challenges for urban areas. Efficient solutions for increasing the environmental sustainability of modern cities have become a top priority for the quality of life of their inhabitants. In our previous research [1,2,3,4,5,6], we proposed an innovative and effective solution for this problem. In detail, we designed and manufactured a prototype platform able to reuse the organic fraction of the municipal solid waste (OFMSW) to produce inertized and valorized panels and artifacts of different shapes. The addition of melamine-formaldehyde based resin to OFMSW gave the most efficient inertization and valorization of the waste [4]. Compared to the urea-formaldehyde (UF) resin, melamine-formaldehyde (MF) resin improved the mechanical performance pre and post artificial weathering and mitigated the human health hazard by reducing the formaldehyde release.

The POIROT (Italian acronym of dOmotic Platform for Inertization and tRaceability of Organic wasTe) is a prototype equipment which provides a conversion procedure for the transformation of organic wastes into a completely inert and reusable material [4]. The transformation process implemented in POIROT is divided into five sub-processes (i.e., pretreatment, valorization, manufacture, control, and identification); each of them has specific functions that are detailed below.

(1) Pre-treatment: to shred, mix, press, filter and eject the treated OFMSW;
(2) Valorization: to transform the OFMSW into stabilized material by integrating the chosen additives into the pre-treated OFMSW;
(3) Manufacture: to deliver the intermediate product by plastic molding and subsequent auto-cleaning of the machinery internal components;
(4) Control: to set and monitor the processing parameters for each sub-process (temperature, residence time, etc.);
(5) Identification: to trace the products by radio-frequency identification (RFID).

Diagnostics were performed throughout the entire transformation process by means of sensors monitoring the physical-chemical properties of the pristine organic waste, the intermediate product and the waste water [2,4]. In the valorization sub-process, the pre-treated OFMSW was modified by the addition of a melamine-formaldehyde based resin that conferred suitable physical and mechanical properties on the recycled panels and, thus, allowed an effective reuse and recycling of the organic waste. Melamine-formaldehyde resin provides several beneficial properties, such as high crosslinking density, water solubility (for organic-solvent-free processing), high strength, cost-effectiveness, rapid curing, high durability, and negligible toxicity hazard thanks to very low formaldehyde release [4].

As explained in a previous work [7], the arising environmental hazards caused by common waste management methods, the decreased space of landfills and the exponential increase in marine pollution require alternative solutions for waste disposal [8]. As a consequence, recycling of wastes is nowadays rapidly growing as it is assumed to be the best approach for the reduction of environment pollution caused by landfill. In particular, compared to traditional disposal processes [9] which involved the incineration, landfill [10,11], or composting [12] of the waste, the proposed technology allows converting a waste to a source for the production of recycled panels. Based on their durability, OFMSW/melamine-formaldehyde recycled panels appear to be good candidates for outdoor applications [13], where they could replace lumber or plastic composites in residential outdoor products such as decks and furniture [4,14,15]. Moreover, the use of melamine-formaldehyde matrix itself can involve a good response in terms of flame resistance [16,17], and outdoor applications need further improvements in terms of both flammability and smoke release. In the U.S., more than 70,000 communities adjacent to wildland vegetation (Wildland Urban Interface, WUI) are at risk of being involved in wildfires [18]. Reduced flammability recycled panels may be beneficial in WUI communities in outdoor applications. For example, they could be used as replacement for wood fencing, which is of main concern in WUI communities because (1) it promotes rapid flame spread and (2) generates embers that in turn can lead to spot fire ignition [19]. The OFMSW recycled panels could also be used to replace medium-density fiberboard (MDF) in indoor applications where an assessment of the fire performance is required due to fire hazard considerations.

In this work, we investigated the effect of fire retardants on the fire response and mechanical performance of the recycled panels to investigate their possible use in the aforementioned applications [20]. Additionally, the effect of accelerated aging on the mechanical properties of the panels was investigated by boiling treatments.

## 2. Materials and Methods

The OFMSW is an unsorted food waste also called, “wet waste” (mainly composed of kitchen waste—fruit, vegetables, meat, fish, bread, coffee grounds, tea bags, etc.—and some garden waste—grass, leaves, very small sprigs, wood ash, etc.) collected according to the Italian standard regulation CER code 200108. In this work, we used OFMSW produced at a local restaurant, which contained food waste and small amounts (less than 0.5% by weight) of soft waste, such as tissues or napkins.

Based on our previous research [1,2,3,4], a melamine-formaldehyde powder polymer (SADECOL P656 manufactured by Sadepan Chemical Srl, Viadana, Italy)—obtained by the condensation between melamine and formaldehyde and modified by the addition of fillers, additives, and hardeners—was used in this work for the inertization of the treated OFMSW. The resin powder, before the cure process, has a formaldehyde mass fraction that is lower than 0.1% [21].

A catalyst (Fast sad SD 10, supplied by Sadepan Chemical Srl, Viadana, Italy) was added to the resin at a mass fraction of 10% in order to reduce the time and temperature of the curing process. In the remainder of the manuscript, the acronym MF (melamine-formaldehyde) is used to refer to the catalyzed resin. In the fire retardant blends, mono-ammonium phosphate (PFR), which may contain several impurities and is usually used as fertilizer (supplied by Sadepan Chemical Srl), or aluminium trihydrate (ATH, supplied by Sibelco (Antwerp, Belgium)) were added as fire retardant agents to the MF/OFMSW blends by properly reducing the MF-resin content, as reported in Table 1.

Table 1 shows the identifier and composition used for each blend. Specifically, the blends are identified as “OFx_MF_yFR”, where “OF” stands for OFMSW, “MF” is the catalyzed resin melamine-formaldehyde, and “FR” (fire retardant) stands for either “PFR” or “ATH”; “x” and “y” indicate the mass fraction % of OF and FR, respectively. The OFMSW content reported in Table 1 refers to the pristine wet OFMSW with a water content of about 70% by mass. Water was later removed by evaporation during the transformation process and curing [4]. A mass fraction of MF between 40% and 50% was used in the blends for manufacturing the panels.

**Figure 1:**
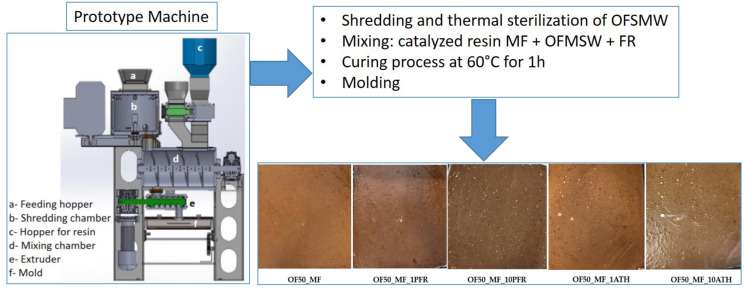
Representative scheme of the production of the experimental panels and pictures showing the top surface of each realized panel.

Multiple panels with compositions reported in Table 1 were produced by using the POIROT (Italian acronym of dOmotic Platform for Inertization and tRaceability of Organic wasTe) prototype [1,2] (Figure 1).

The specimens for mechanical testing, boiling testing, and cone calorimetry were obtained by cutting the panels to size. Prior to testing, all samples were conditioned at a relative humidity (RH) of 65% ± 2%, and temperature of 20 ± 3 °C until the mass of the samples stabilized within 0.1% by mass.

Flexural tests were carried out on specimens with nominal dimensions of 10 × 1 × 0.5 cm^3^ by using 3-point bending configuration and a dynamometer (model Lloyd LR5 K, Lloyd Instruments Ltd., Bognor Regis, West Sussex, UK) equipped with a load cell of 1 kN according to the UNI EN 310 regulation [22], which implements the European directives on cured panels for building applications. Six samples were tested for each blend reported in Table 1. The crosshead speed was 1.5 mm/min. Samples failure occurred within 60 s.

Boiling tests were performed on samples 10 × 1 × 0.5 cm^3^ according to UNI EN 1087-1 standard [23]. Briefly, the specimens were immersed in neutral water at 20 °C, followed by heating in an oven at 110 °C until water boiling started. Then, the samples were kept in boiling water for additional 120 min before being removed from the oven. After cooling, flexural tests were immediately carried on the samples without any further drying according to the procedure described above. For each blend, six replicate tests were performed. The six samples were extracted from the different panels in order to account for variation in properties due to heterogeneity in OFMSW and panel-to-panel properties variations due to processing.

A cone calorimeter (Fire Testing Technology, East Grinstead, West Sussex UK) was used to measure the ignition characteristics, heat release rate, total heat release and smoke yield according to the ASTM E1354 standard [24]. An external heat flux of 50 ± 0.5 kW/m^2^ was applied. The distance between the cone heater and the top surface of the specimen was 60 mm. A 10 kV electric spark igniter was used as the ignition source. The samples had nominal dimensions of 10 × 10 × 0.5 cm^3^ and were placed in an aluminum catch pan. The specimen/pan assembly was then placed on a ceramic wool insulator and it was covered with a standard retainer frame, as described by ASTM E1354 standard, which prevented edge burning of the specimen and exposed only a surface area of 88.4 cm^2^ over the top of the specimen.

The thermal stability of the samples with and without the fire retardant was assessed by Thermo-Gravimetric Analysis (TGA), with a TA Instrument SDT Q600 (manufactured by TA Instrument, New Castle, DE, USA). The samples were heated in an alumina holder, from 20 °C up to 800 °C at a heating rate of 10 °C/min under air atmosphere; at least three measurements were performed on each sample.

Infrared spectra and X-ray diffraction (XRD) analysis was carried out on the char obtained after cone calorimeter tests. In detail, the infrared spectra were recorded in the wavelength range between 4000 cm^−1^ and 400 cm^−1^, using a Fourier Transform Infrared spectrometer (FTIR) Jasco 6300 (manufactured by Jasco International CO., Tokyo, Japan), and a KBr round crystal window was used. Each measurement was obtained with 128 scans and 4 cm^−1^ of resolution. XRD analyses (manufactured by Rigaku, Tokyo, Japan) were carried out with CuKα radiation (λ = 1.5418 Å) in the step scanning mode recorded in the 2θ range between 10 °C and 40 °C, with a step size of 0.02 °C and step duration equal to 0.5 s.

## 3. Results and Discussion

**Figure 2:**
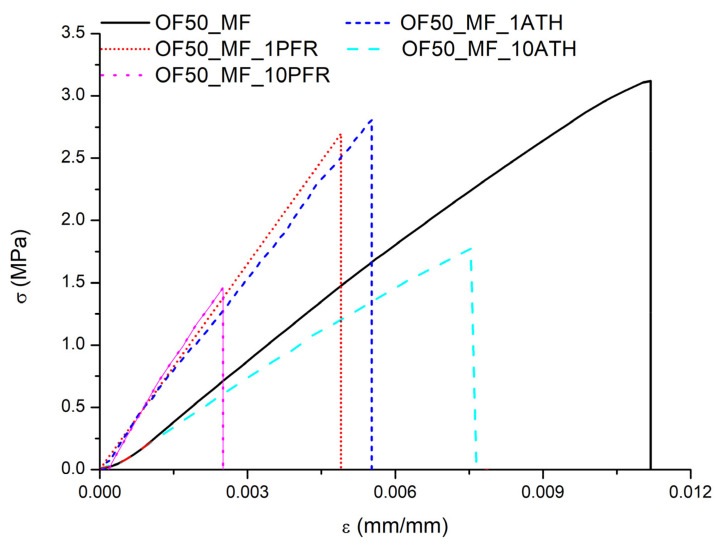
Stress/strain curves of the control and modified panels.

Type A uncertainties are reported for all data as one standard deviation calculated over three independent observations, unless otherwise stated [25]. Representative stress (σ)-strain (ε) curves measured in the flexural tests are shown in Figure 2. 

The values of ultimate strength *(σ_max_*), strain at break *(ε_max_*), and modulus (*E*) for each sample type are reported in Table 2. 

The addition of fire retardants induced a decrease in *σ_max_* (in detail, 47% for OF50_MF_1PFR, 55% for OF50_MF_10PFR, 20% for OF50_MF_1ATH, and 47% for OF50_MF_10ATH panel) and in *ε_max_* (54% for OF50_MF_1PFR, 53% for OF50_MF_10PFR, 46% for OF50_MF_1ATH and 22% for the OF50_MF_10ATH panel) of the material compared to the OF50_MF. This result can be attributed to the defects caused by an inhomogeneous dispersion of the additive, poor adhesion between the resin and the fire retardants, and the reduction in the amount of cross-linker MF when fire retardants are added.

An increase in E was found with either fire retardant. At a 1% mass fraction of fire retardant, the elastic modulus increased up to 333 ± 143 MPa (about 40% average increase) with PFR and up to 439 ± 96 MPa (about 70% average increase) with ATH as compared to OF50_MF. When the fire retardants were added at a mass fraction of 10%, the elastic modulus increased further with PFR from 333 ± 143 MPa to 404 ± 189 MPa but decreased with ATH from 439 ± 96 MPa to 267 ± 175 MPa.

**Figure 3:**
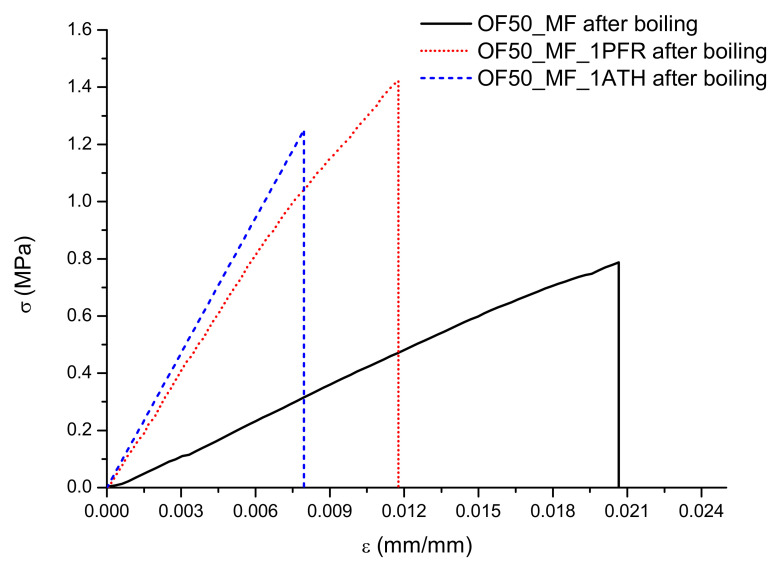
Stress/strain curves after the boiling treatment.

Additional flexural tests were carried out with the specimens after the boiling treatment. Test results are reported in Figure 3 and Table 3. Results at a 10% mass fraction of fire retardants are not shown because the specimens disintegrated in water during the boiling treatment and could not be tested.

As in many composites, water has a plasticizing effect on the composites, increasing elongation while reducing strength. The control formulation OF50_MF showed a 4-fold decrease in flexural strength and a 6-fold decrease in elastic modulus. In comparison, specimens with a 1% mass fraction of either ATH or PFR were less affected by the boiling treatment: OF50_MF_1PFR had no significant decrease in ultimate strength, while OF50_MF_1ATH had a 2-fold reduction in ultimate strength; both formulations showed approximately a three-fold reduction in flexural modulus. The best performers after boiling treatment were: OF50_MF_1PFR and OF50_MF_1ATH in terms of σ*_max_* (about 1.3 MPa), OF50_MF_1ATH in terms of E (about 160 MPa), and OF50_MF in terms of ε*_max_* (about 23 × 10^−3^). OF50_MF_1PFR also offered the highest toughness (as pointed out by the integral of the stress–strain curves) and ultimately provided the best combination of toughness, strength, and stiffness between the tested formulations after boiling treatment.

**Figure 4:**
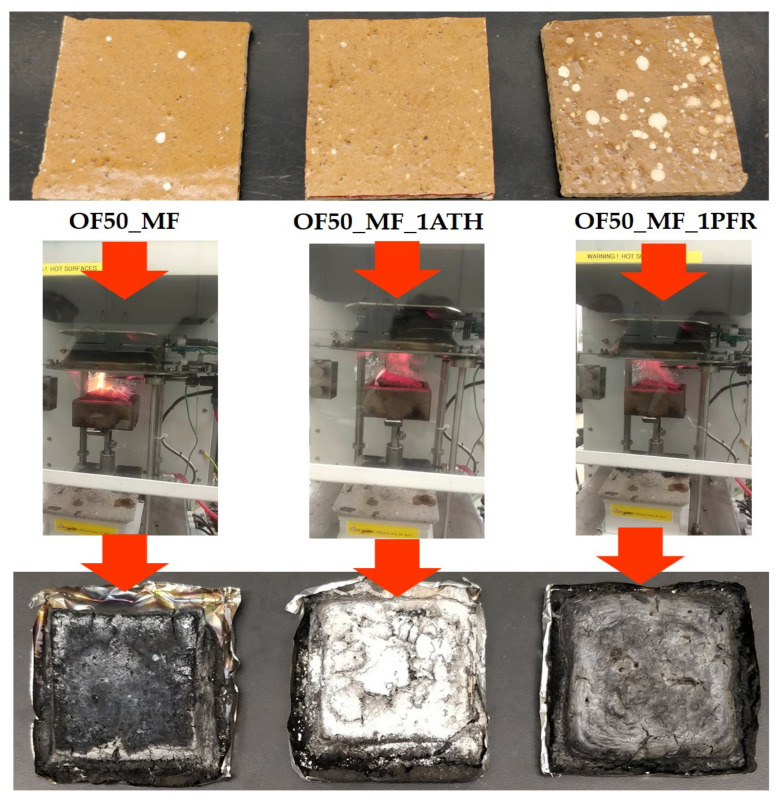
Samples before (top), during (about 300 s from test start) (center), and after the cone calorimeter test (bottom).

Cone calorimeter tests were performed in triplicates, except OF50_MF_10PFR, which was run only once because its high brittleness prevented a proper sample preparation. Figure 4 shows pictures of the specimens OF50_MF, OF50_MF_1PFR, and OF50_MF_1ATH before testing, during testing (about 300 s from test start), and after testing. As seen in Figure 4, the addition of ATH at 1% mass fraction was able to decrease the flame size compared the control specimen and the addition of PFR at 1% mass fraction fully suppressed ignition. The addition of fire retardants also appeared to visibly increase smoke production, presumably due to the increase in unburnt pyrolyzates which were released. The residues of the specimens show a small amount of char left for OF50_MF, a typical white residue due to the generation of alumina (produced by the thermal decomposition of ATH) [26] for OF50_1ATH, and a slightly intumescent carbonaceous residue for OF50_1PFR.

**Figure 5:**
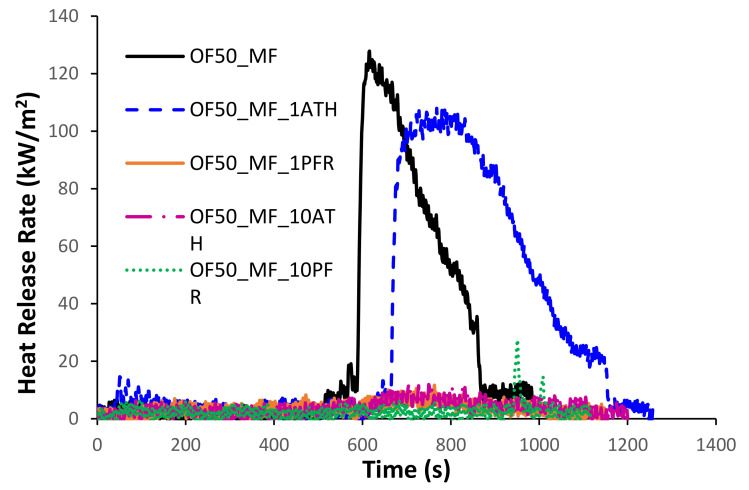
Representative heat release rate (HRR) curves measured by cone calorimetry.

Figure 5 shows the representative heat release rate (HRR) curves measured by cone calorimetry. The control formulation OF50_MF had a single peak of HRR (PHRR) of over 120 kW/m^2^ at about 600 s. The addition of 1% ATH did not have a major impact on fire response (see OF50_MF_1ATH). All other formulations showed a drastic reduction in peak of heat release rate.

Cone data are summarized in Table 4, which reports the time of ignition (TOI), the total heat released (THR), the peak of HRR (PHRR), the time to PHRR (TPHRR), and the effective heat of combustion (EHC). The values of THR and EHC were calculated throughout the entire test.

On one hand, the addition of 1% mass fraction of ATH did not improve the fire performance of the control formulation and actually slightly increased the THR from 22 ± 4 MJ/m^2^ to 36 ± 1 MJ/m^2^ (see OF50_MF and OF50_MF_1ATH in Table 1); on the other hand, the addition of 1% mass fraction of PFR prevented ignition in all three tested specimens and decreased the THR to 6 ± 2 MJ/m^2^. Surprisingly, when the mass fraction of PFR was increased from 1% to 10% in OF50_MF_10PFR, ignition occurred after 950 s (single test); however, flaming was short lived, and this formulation had the lowest THR (2 MJ/m^2^) and highest residue (52%). Besides OF50_MF_1PFR, all triplicate tests for OF50_MF_10ATH also did not ignite; this formulation also showed the second lowest THR value of 7 ± 2 MJ/m^2^. The PHRR was about 120 kW/m^2^ in the control formulation, remained roughly the same in OF50_MF_1ATH, and drastically decreased (below 30 kW/m^2^) in all other formulations.

In terms of smoke generation, the control sample OF50_MF had a value of 3.0 ± 0.1 m^2^ that was not significantly different from the values of 2.3 ± 0.7 m^2^ measured for OF50_MF1ATH and 3.1 m^2^ (single test) measured for OF50_MF10PFR; however, the smoke generation increased in specimen OF50_MF1PFR with a value of 27.6 ± 7.5 m^2^ and OF50_MF10ATH with a value of 26.0 ± 5.4 m^2^.

Ultimately, cone data revealed that the control formulation with a PHRR of about 120 kW/m^2^ and an EHC of about 5 MJ/kg had an intrinsically low flammability. For reference, red cedar—which is a wood type commonly used in USA for fencing—has an EHC of about 11 MJ/m^2^ measured by micro combustion calorimetry and a PHRR of about 190 kW/m^2^ measured by cone calorimetry at an external heat flux of 50 kW/m^2^ [27]. At the same 50 kW/m^2^ heat flux in the cone calorimeter, Hasburgh et al. [28] reported values of PHRR and THR for MDF-based panels of about 250 kW/m^2^ and 170 MJ/m^2^, respectively; White et al. [14] reported values of PHRR between 176 and 249 kW/m^2^ and values of THR between 92 and 329 MJ/m^2^ for various types of wood species; White et al. [14] also reported values of PHRR between 374 and 1790 kW/m^2^ and values of THR between 171 and 609 MJ/m^2^ for various types of wood plastic composites.

These data indicate that the flammability of the pristine OFMSW/melamine-formaldehyde panels is already significantly lower than MDF panels, lumber, and wood plastic composites. More importantly, the addition of only 1% mass fraction of PFR allowed manufacturing panels that did not ignite at an external heat flux of 50 kW/m^2^. Thus, OFMSW/melamine-formaldehyde panels might indeed represent a valid sustainable approach to reduce the fire hazard in indoor and outdoor applications by replacing materials like wood, MDF panels, and wood plastic composites.

The fire performance of lumber can be temporarily improved by using fire retardant coatings but the effect of the coatings has been found to be negligible after just a few months due to weathering [27,29]; thus, a material with an intrinsically low flammability like the OFMSW recycled panels might be preferable in outdoor applications. As shown above, the fire performance of OFMSW panels can be further improved by fire retardants. Even if we did not measure the effect of weathering on the fire-retardant panels, their fire performance is not expected to be affected by weathering as much as in fire retardant coated wood, where cracking, peeling, and leaching in the fire retardant coating can lead to a drastic and rapid decrease of the fire performance within few months [27,29].

Table 4 also shows the values of residues measured by TGA in air. The residue was about 4% by mass in the control formulation. In presence of fire retardants, the residue increased due to charring reactions promoted by PFR or the inorganic residue generated by the decomposition of ATH.

**Figure 6:**
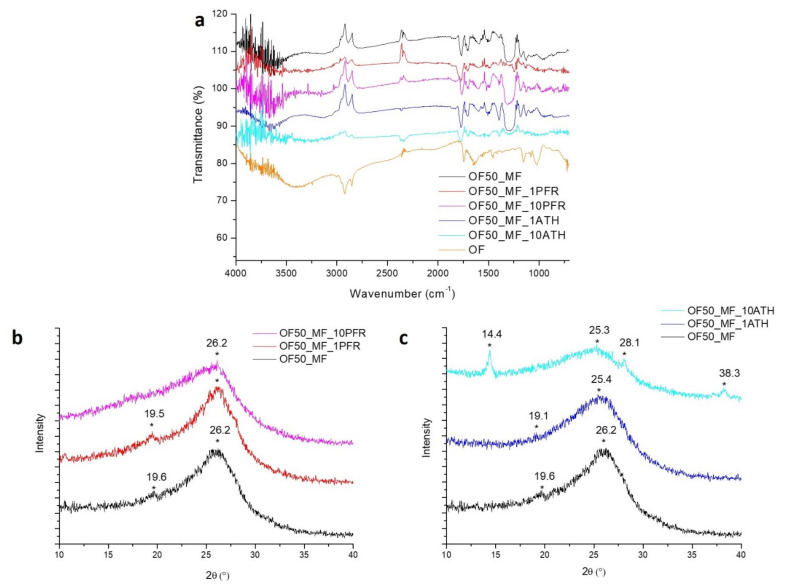
Analyses of residues from cone calorimetry: (**a**) FTIR spectra; (**b**,**c**) XRD spectra, where * indicates the principal diffrattometric peaks.

FTIR and XRD analyses were carried out on the residues collected from the samples after cone calorimetry in order to gain insights into the chemical structure of the residue (see Figure 6). FTIR spectra (Figure 6a) showed multiple peaks that are characteristic of both OF and MF, indicating that only partial decomposition of the sample occurred, and even without the fire retardant, the chemical structure of the material was partially preserved. In particular, CH stretching (about 29,000 cm^−1^), C=O stretching (1710 cm^−1^), C=C stretching (1650 cm^−1^), and C–O stretching (1150 cm^−1^) that were detected in all the samples after burning, can be attributed to residual OF moieties in the char. FTIR spectra for all residues also show typical peaks attributed to MF resin [30], such as the N–H stretching frequency of secondary amine at 3500 cm^−1^, methylene C–H bending vibration at 1456 cm^−1^, C–N stretching at 1350 cm^−1^ and a broad peak at 1290 cm^−1^, due to C–O stretching.

The XRD patterns (Figure 6b,c) from the residues indicated a prevalently amorphous structure for all specimens. All residues contained graphite-like structures as indicated by the presence of a band at 2θ around 25–26 °C [31]. In the residue for OF50_MF_10ATH, a few weak crystalline XRD peaks at 14.4 °C, 28.1°, and 38.3 °C were evident; they were attributed to the presence of residual ATH and/or the formation of alumina [32].

## 4. Conclusions

The influence of two fire retardants, mono-ammonium phosphate (PFR), and aluminum trihydrate (ATH) on the mechanical properties, durability, and fire resistance of OFMSW-based panels was assessed. The addition of low amounts (1% mass fraction) of both PFR and ATH produced an increase in elastic modulus of about 40% and 70%, respectively, but also significantly decreased ultimate strength (20% reduction in OF50_MF _1ATH and 47% reduction in OF50_MF _1PFR) and elongation at break (46% reduction in OF50_MF _1ATH and 54% reduction in OF50_MF _1PFR). The addition of a higher content of fire retardants (10% mass fraction) increased the brittleness of the samples and made handling and processing of the material problematic. The brittleness of the samples further increased after artificial accelerated weathering carried out by a boiling treatment; however, the samples with 1% mass fraction of ATH or PFR still retained good mechanical properties.

After boiling treatment, the values measured for ultimate strength and elastic modulus in OF50_MF _1ATH and OF50_MF _1PHR were 2 to 4 times higher than those measured for the control OF50_MF.

Overall, OF50_MF _1PHR provided the best combination of toughness, strength, and stiffness between the tested formulations after boiling treatment.

In terms of fire response, the addition of only 1% mass fraction of PFR allowed to prevent ignition in the cone calorimeter at an external heat flux of 50 kW/m^2^; this was not the case for ATH. On the other hand, the addition of fire retardants was found to increase the production of smoke; specifically, a smoke generation comparable to the sample without flame retardant was found for OF50_MF1ATH, while higher smoke generation was detected with the addition of 1% of PFR and 10% of ATH.

Ultimately, the formulation OF50_MF _1PFR provided an optimal balance of mechanical performance, durability, and fire performance (within the formulations investigated in this study); such a material might be potentially used to replace wood, wood composites, and MDF panels in applications where superior fire performance is required.

## Figures and Tables

**Table 1:** Composition of the blends.

Blend	OFMSW (Mass Fraction %)	Catalyzed Resin, MF (Mass Fraction %)	Fire Retardant (Mass Fraction %)
OF50_MF	50	50	-
OF50_MF_1PFR	50	49	1
OF50_MF_10PFR	50	40	10
OF50_MF_1ATH	50	49	1
OF50_MF_10ATH	50	40	10

**Table 2:** Values of ultimate strength (σ_max_), strain at break (ε_max_), and modulus (E) measured by flexural tests. Uncertainties are reported for all data as one standard deviation calculated over six independent observations.

Sample	*σ _max_* (MPa)	*ε_max_* (10^−3^ mm/mm)	E (MPa)
OF50_MF	2.79 ± 0.41	10.0 ± 2.0	236 ± 42
OF50_MF_1PFR	1.48 ± 0.55	4.6 ± 0.55	333 ± 143
OF50_MF_10PFR	1.24 ± 0.21	4.7 ± 1.6	404 ± 189
OF50_MF_1ATH	2.21 ± 0.53	5.4 ± 0.9	439 ± 96
OF50_MF_10ATH	1.46 ± 0.65	7.8 ± 1.1	267 ± 175

**Table 3:** Mechanical properties of the samples after the boiling treatment. Uncertainties are reported for all data as one standard deviation calculated over six independent observations.

Sample after Boiling Treatment	σ_*max*_ (MPa)	ε*_max_* (10^−3^⋅mm/mm)	E (MPa)
OF50_MF	0.67 ± 0.14	23 ± 6	37.7 ± 4.4
OF50_MF_1PFR	1.34 ± 0.42	16 ± 3	110.4 ± 32.7
OF50_MF_1ATH	1.35 ± 0.20	10 ± 1	161.4 ± 20.3

**Table 4:** Cone calorimetry data and Thermo-Gravimetric Analysis (TGA) residue.

Sample	TOI (s)	THR (MJ/m^2^)	PHRR (kW/m^2^)	TPHRR (s)	EHC (MJ/kg)	Smoke (m^2^)	Cone Residue (Mass Fraction %)	TGA Residue (Mass Fraction %)
OF50_MF	546 ± 172	22 ± 4	122 ± 14	595 ± 162	4.9 ± 1.5	3.0 ± 0.1	32.2 ± 1.4	3.7 ± 0.1
OF50_MF_1PFR	n/a ^ξ^	6 ± 2	12 ± 3	777 ± 58	1.0 ± 0.2	27.6 ± 7.5	31.4 ± 7.2	8.4 ± 0.4
OF50_MF_10PFR	931 *	2 *	27 *	950 *	0.4 *	3.1 *	52.3 *	12.6 ± 0.3
OF50_MF_1ATH	720 ± 59	36 ± 1	122 ± 20	800 ± 28	6.2 ± 0.2	2.3 ± 0.7	32.1 ± 2.8	9.2 ± 0.2
OF50_MF_10ATH	n/a ^ξ^	7 ± 2	24 ± 16	780 ± 37	1.2 ± 0.3	26.0 ± 5.4	36.7 ± 6.4	19.6 ± 0.8

* Single cone calorimeter test. ^ξ^ Not available, no ignition.

## Data Availability

Part of this work was carried out by the National Institute of Standards and Technology (NIST), an agency of the US government and by statue is not subject to copyright in USA. The identification of any commercial product or trade name does not imply endorsement or recommendation by NIST. The policy of NIST is to use metric units of measurement in all its publications, and to provide statements of uncertainty for all original measurements. In this document, however, data from organizations outside NIST are shown, which may include measurements in non-metric units or measurements without uncertainty statements.
